# Medical decision‐making under risk and uncertainty: Anesthetists' decision to proceed with surgery

**DOI:** 10.1111/risa.70027

**Published:** 2025-05-21

**Authors:** Zijing Yang, Yaniv Hanoch, Zvi Safra, Tigran Melkonyan, Olivera Potparic, James Palmer

**Affiliations:** ^1^ Behavioural Science Group, Warwick Business School University of Warwick England UK; ^2^ Interdisciplinary Centre for Social Sciences (ICSS) Zhejiang University China; ^3^ University of Wolverhampton Wolverhampton UK; ^4^ Culverhouse College of Business University of Alabama Tuscaloosa Alabama USA; ^5^ Chelsea and Westminster NHS Foundation Trust London UK

**Keywords:** anesthesia, experience, gender, regret, risk preference, uncertainty

## Abstract

There is a paucity of work examining anesthetists' willingness to proceed as attending anesthetists (hereafter, WTP) in response to different risky medical conditions. Earlier studies offer only a partial and indirect explanation as to why variations in WTP exist. We evaluated whether psychological factors of risk‐taking tendencies, attitudes toward uncertainty, sense of regret, and demographic variables, particularly experience and gender, might clarify the disparities in an anesthetist's WTP. Anesthetists from two National Health Service Trusts in England (i.e., hospitals) viewed, in random order, three different realistic scenarios (representing low‐, medium‐, and high‐risk cases) and were asked to indicate how likely they were to agree to proceed as the attending anesthetist. They also answered questions evaluating their risk‐taking tendencies, comfort with uncertainty, and tendency to experience regret. Anesthetists varied in their WTP. Importantly, our data revealed that a sense of uncertainty and regret, but not a risk attitude, could help explain these variations. Female anesthetists were less likely to agree to proceed as attending anesthetists regardless of the level of risk or individual differences, but we found no relationship between levels of experience and WTP. Examining anesthetists' WTP in isolation provides an important but only partial picture. Gaining a better understanding of the factors that drive decision‐making is vital for improving both training and practice. In particular, given the high proportion of women in anesthesia, the gender difference found in this study has important implications for anesthetic training and practice.

## 1 INTRODUCTION

Anesthesia is a prominent field of specialization within the UK (National Health Service [NHS], 2015) and the US (Association of American Medical Colleges [AAMC], 2023) healthcare system. As one of the most high‐risk specialties within the medical field (Adams & Smith, 2001), anesthetists are frequently asked to make risky operation‐related decisions. Indeed, within the UK healthcare system, anesthetists' judgments or decisions are often taken at face value, even if it means not operating on a patient. They play crucial roles in leading the clinical management of intensive care units and collaborate closely with a wide spectrum of other medical specialists, such as cardiac arrest teams and obstetrics. They provide essential medical care before, during, and after the operation, which makes them “the most important member of the medical team for patient's safety and care” (Verma et al., 2015). However, despite the clinical significance of their decisions, there is a limited understanding regarding the observed extent of variability of these decisions and the factors that impact these variations.

Previous research has revealed significant disparities in anesthetists' decisions when determining whether to proceed with a surgical procedure or pursue nonoperative management, even in cases where medical guidelines provide clear recommendations (Alkassimi et al., 2020; Greig et al., 2017). The causes of unexplained variations, that is, variations that are not brought on by patient differences, have not yet been fully examined. One possible explanation for variations in decisions could rest with variations in personal traits. Indeed, a large corpus of data has examined how personal traits impact medical decisions in a range of medical specialties and treatments (Wu et al., 2021).

Tendency to experience regret, tolerance to uncertainty, and risk preference representing three personal traits have been documented to affect medical decisions. Regret has been shown repetitively in textual responses as one of the reasons affecting anesthetists' decision to proceed with an operation (Greig et al., 2017). Others have reported that regret impacts physicians' decision of whether or not to serve on a patient (Bagante et al., 2016; Cucchetti, et al., 2015), and in making future treatment decisions (Müller et al., 2020). Tolerance of uncertainty and risk preference have been shown to play a significant role in a wide range of medical decision‐making. For instance, physicians with low tolerance of uncertainty demonstrate lower willingness to recommend new medical interventions (Strout et al., 2018), difficulties in selecting the best treatment (Bursztajn, 1981), and risk‐seeking physicians tend to request fewer tests to patients (Tubbs et al., 2006). Nevertheless, there is a lack of systematic investigation into the effects of tolerance of uncertainty and risk preference on anesthetists' operation‐related decisions.

Additionally, research has revealed that the anesthetists' gender can impact medical decision‐making during their training (Pearce et al., 2020). However, despite the tendency of females to be more risk‐averse than males, there is very limited data regarding the role gender plays in anesthetists' operation‐related decisions.

This article extends earlier work by examining the impact of personal traits on anesthetists' willingness to proceed as attending anesthetists in different risky surgical scenarios. We conducted an online study to elicit individual preferences, including risk preference, tolerance of uncertainty, and tendency to experience regret, as well as collect demographic and experience information. Our unique gender‐balanced sample consists of anesthetists working in the UK. Our research makes three primary contributions. First, we provide evidence that low tolerance of uncertainty and a tendency to experience regret have significant negative effects on the WTP in all risky surgical scenarios. Second, we provide novel insights into gender differences in physicians' clinical decision‐making and risk‐taking. Specifically, we show that female anesthetists are significantly less likely to agree to participate in an operation in all risky scenarios compared to male anesthetists. Finally, our data reveal that experience and risk preference are not significant factors in the WTP. This contrasts with previous research that has shown that an increase in experience correlates to a greater willingness to take more risks (Greig et al., 2017). A better understanding of the impact of tolerance for uncertainty, the tendency to experience regret and risk preference, and the effect of gender on operation‐related decision‐making could have important ramifications for clinical education and clinical practice.

## 2 LITERATURE REVIEW

Variations in anesthetists' decisions to serve as attending physicians can be found in the literature. Greig et al. (2017) presented anesthetists with 11 risky scenarios and asked whether they would proceed with the medical procedure, that is, a go/no‐go decision. Even in clinical scenarios where national guidelines would indicate that an operation should be canceled, there was never consensus regarding whether to proceed. A similar result was reported even in cases where the medical guidelines unambiguously suggested that the surgery should be canceled (Alkassimi et al., 2020). The unexpectedly high variability called for the attention of anesthesia professionals (i.e., for those working as anesthetists), who wanted to avoid situations in which similar patients receive different treatments, presumably leading to some nonjustified treatments (Ehrenfeld & Fleischut, 2017).

Regret is a common response among both surgeons and patients (Wilson et al., 2017). The study by Greig et al. (2017), mentioned above, asked anesthetists to provide a rationale in free‐text responses for their go/no‐go decision. The role of regret was found to be associated with the decision to proceed, while experience correlated to a greater willingness to take on more risk. Feeling regret is common because adverse outcomes may occur even if the best decisions and actions are followed (Bacon, 1990; Boyle et al., 2020; Cheval et al., 2019; Courvoisier et al., 2011; Wilson et al., 2017). Two systematic reviews of the medical literature have shown that regret is involved in a host of medical decisions (Becerra Pérez et al., 2016; Wilson et al., 2017). Others have reported that regret is linked to physicians' employing a maximizing decision strategy. Interestingly, using a maximizing strategy is associated with the tendency to examine more alternatives and seek the best option, which often leads to an even greater feeling of regret (Djulbegovic et al., 2015), which impacts diagnostic and treatment decisions (Wilson et al., 2017). Previous research has indicated that elevated levels of regret have been associated with heightened emotional distress and self‐blame, resulting in decision‐making that negatively impacts patient outcomes (Engel et al., 2006), as well as affecting physicians shared decision‐making strategies (Speck et al., 2016). Cucchetti et al. (2015) and Bagante et al. (2016) found that surgeons were more likely to regret a decision of nonoperative intervention versus operative intervention by measuring physician regret directly on a case‐by‐case analysis. Regret, thus, plays an important role in physicians' decisions, whether in the decision strategy employed, their mental well‐being, and whether or not to go ahead with a procedure.

In addition to regret, tolerance of uncertainty in medical decision‐making captures three broad categories: complexity, which arises from difficulty in comprehending information, risks which arises from the indeterminacy of future outcomes, and ambiguity, which arises from limitations in the reliability of information (Han et al., 2015). This phenomenon is exacerbated by the continuous introduction of new medical technology, which outpaces the development of evidence regarding their benefits, harms, and implications (Hillen et al., 2017). As a result, its relationship with various health and healthcare‐related outcomes has become the focus of an expanding body of empirical research. Tolerance of uncertainty is associated with significant difficulties in selecting the best course of action (Bursztajn, 1981; Fox, 1980), increased levels of anxiety (Johnson et al., 1988), and fear of malpractice litigation (Gutheil et al., 1984). It is also correlated positively with adverse medical outcomes and deterioration in patient confidence (LaMartina et al., 2018; Ogden et al., 2002; Tubbs et al., 2006), increased support for shared decision‐making (Kannan et al., 2020), and increased overconfidence bias among surgeons (Teunis et al., 2016). A systematic review by Strout et al. (2018) focused on the effect of tolerance of uncertainty on healthcare outcomes. In general, lower uncertainty tolerance was associated with higher psychological distress and burnout among healthcare providers. Tolerance of uncertainty has also shown associations with behavioral outcomes, including clinician willingness to recommend new medical interventions. As medical practice involves high levels of uncertainty, the literature summarized above indicates that higher tolerance of uncertainty is a predictor of better outcomes both for patients and physicians.

A parallel body of research has focused on the association between risk‐taking tendencies and clinical practices. Work by Gurmankin et al. (2004) has reported that physicians' risk preferences were highly variable relative to the stated risk, which may be attributable to differences in individual risk preferences. Importantly, variation in risk preference has been used to explain differences in clinical practice. Compared to risk‐seeking physicians, for example, risk‐averse ones are more likely to admit patients with acute chest pain, use computed tomography coronary angiography (Pines et al., 2010), as well as using imaging technology for emergency department patients with abdominal pain (Pines et al., 2009). Using a survey of a large cohort of surgeons, Sacks et al. (2016) found that the likelihood of operating decreases (increases) with the risks (benefits) perceived by surgeons. These authors also uncovered a significant variation in the surgeons' evaluation of the risks and benefits of operating and, even more importantly, in their decision whether to serve. Tubbs et al. (2006) found that both risk attitudes and reactions to uncertainty influenced surgeons' decisions. For example, among patients evaluated in the emergency room for chest symptoms, risk‐seeking physicians admitted significantly fewer patients who did not have acute myocardial infarction than risk‐averse physicians. Taken together, the evidence presented above suggests that risk aversion among physicians is associated with a more conservative clinical approach.

Another branch of literature explored the role of gender in medical decision‐making. Champagne‐Langabeer and Hedges (2021) conducted a systematic review of the literature on gender differences in the clinical decision‐making of physicians and discovered significant differences in disease diagnosis, disease treatment, and treatment outcomes. Schneider et al. (2010), likewise, found that anxiety among male general practitioners (GPs) in response to uncertainty was negatively related to diagnostic reasoning and clinical judgment—one of the essential skills for physicians (Croskerry, 2009)—while the association was positive among female GPs. In other domains of medicine, researchers have reported that male physicians tend to adopt new technologies, such as new prescriptions, faster than female physicians (Méndez et al., 2021). Investigations examining medical care for type 2 diabetes have reported that female physicians provided better overall care (Berthold et al., 2008). When it comes to pain management, some reports suggest that female physicians prescribe lower doses compared to their male counterparts (Weisse et al., 2003), and additional research has shown that male obstetrician‐gynecologists are significantly more likely to perform Cesarean section compared to their females' counterparts (Mitler et al., 2000). In the field of anesthesia, Pearce et al. (2020) found significant gender effects in anesthesia training. Male trainees reported 10 times more procedures performed, rated themselves at a higher level of training competency than their actual training levels, exaggerated their procedural experience to supervisors, and felt substantially better prepared for independent practice in their final year. In contrast, females reported greater levels of gender bias among patients. Sidhu and Civil (2019) highlighted that the confidence gap and unconscious bias could be the factors causing gender inequality in anesthesia education. Regarding patients' treatment, the literature suggests that female physicians tend to make less risky decisions, although the quality of their decisions is at times superior to those of their male counterparts.

Based on this body of work, our study was specifically designed to examine whether tolerance of uncertainty, regret, sensitivity, risk preference, and gender impact WTP. Specifically, we predicted the following:

Hypothesis 1 A higher tolerance of uncertainty leads to a greater WTP.

Hypothesis 2 A higher sense of regret leads to a lower WTP.

Hypothesis 3 Different risk preferences lead to different WTP responses.

Hypothesis 4 Being a male (vs. female) will be associated with a higher WTP.

Hypothesis 5 Longer experience will be associated with a higher WTP.

## 3 METHOD

### 3.1 Study design

The participants viewed, in random order, three different realistic scenarios to represent low‐, medium‐, and high‐risk levels of surgery risks (Table 1). The scenarios were developed by an experienced anesthetist consultant (JP)—a term used in the UK NHS to refer to the most senior grade of hospital doctors—and were validated by another 10 experienced anesthetist consultants. The scenarios were selected from a pool of 26 scenarios created by a consultant anesthetist (JP) in 2018. To ensure that all participants viewed and reviewed the same scenarios, two experienced consultant anesthetists (JP and OP) chose the best representative scenarios to use in the study. Another consultant in a different hospital also reviewed these scenarios to ensure their suitability for our survey.

**TABLE 1:** The three scenarios.

Low risk	A 22‐year‐old head injured (a declared brain‐dead patient whose organs are being removed for donor purposes) previously fit and well with a good cardiorespiratory reserve (played rugby until the head injury). The procedure is needed in 2 h' time. For the sake of this case, treat transplant as an area of expertise or interest. Your usual theater/ODP and scrub staff who are well known to you will be helping and the surgeon is known to you.
Medium risk	A 65‐year‐old man with moderate renal dysfunction (eGFR 45) and a BMI of 42 has an exercise tolerance of 4METS (climbs stairs without stopping) and needs elective repair of an inguinal hernia tomorrow. You trained with the surgeon, it is a theater you regularly work in and the ODP & scrub team are known to you. You have not “gassed” a hernia for years as you only do orthopedics.
High risk	A previously well 3‐year‐old is brought into casualty choking. On arrival, you find the child cyanosed, gasping, and bradycardic. Everyone who is not trying to do CPR is panicking, you do not recognize any A&E staff, the parents are crying, and the ODP came down with you because he did not know his way to A&E. As you try to start addressing the main issues, a consultant ENT surgeon you know well arrives and says that immediate tracheostomy is needed and there is no time to go to theater.

The participants reviewed the three scenarios and provided a rating from “Extremely Unlikely” to “Extremely Likely” on a scale from 0 to 100 for four questions on each scenario, and the initial marker is at the 50‐point mark (If a respondent does not want to respond, they have the option to leave the question unanswered and their response will not be included in our data.). The critical decision question was, “For this patient scenario, please indicate how likely you would agree to be their anesthetist.”

The participants then completed several individual measures: two questions from the Nightingale risk preference instrument measure (Nightingale, 1987), a 12‐item Stress from Uncertainty Subscale questionnaire (Gerrity et al., 1990) that measures tolerance of uncertainty, and a 5‐item regret scale questionnaire (Schwartz et al., 2002) that measures a tendency to experience regret. The survey concluded with demographic questions (gender, age, ethnicity, current medical position, number of years practicing anesthesia, and number of operations per year).

### 3.2 Participants

A total of 138 anesthetists from two large‐sized UK teaching hospitals located in two major cities were approached and 96 were included in the final analysis. We inserted the screening question before the survey questions to ensure only anesthetists participated. A total of 96 (69.56%) participants passed the screening question and completed the survey, 20 (14.49%) participants failed this question, and 22 (15.94%) did not finish the survey. The participants were told that three Amazon vouchers worth ₤100 would be randomly given to those who participated in the study. The average time for completing the survey is approximately 15 min. The sample is composed of 56% male and 44% female, and the average age of the participants is 46. According to the Medical Workforce Census Report 2020 from the Royal College of Anaesthetists, women make up 38% of the consultant workforce in the UK. About 39% of the workforce is aged 50 or older. A comparison of these numbers with our sample statistics reveals that our sample reflects the distribution of the anesthetists' workforce in the UK. Most of the participants are consultants (67.7%), while the remaining fall into either trainee (23.2%) or “other” categories. 89.69% of the surveyed individuals take part in more than 100 procedures per year. Most of the participants were white (69.47%), followed by Asian/Asian British and mixed/other ethnic groups (see Table 2).

**TABLE 2:** Summary statistics of demographics.

		frequency	%	cum.%
Gender	Male	54	56.25	56.25
	Female	42	43.75	100.00
Age	25–34	11	12.36	12.36
	35–44	33	37.08	49.44
	45–54	24	26.97	76.40
	55–64	18	20.22	96.63
	65–75	3	3.37	100.00
Experience (years)	Less than 10	32	34.04	34.04
	11–20	32	34.04	68.09
	21–30	19	20.21	88.30
	31–40	11	11.70	100.00
Ethnicity	White	66	69.47	69.47
	Asian/Asian British	18	18.95	88.42
	Mixed/Other ethnic group	11	11.58	100.00
Position	Consultant	65	67.71	67.71
	Trainees	22	22.92	90.63
	Other	9	9.38	100.00
Operations per year	Less than 100	10	10.31	10.31
	101–250	29	29.90	40.21
	251–500	32	32.99	73.20
	Over 500	26	26.80	100.00
Health status	Not healthy/Very unhealthy	5	5.15	5.15
	So‐so	23	23.71	28.87
	Healthy	57	58.76	87.63
	Very healthy	12	12.37	100.00

### 3.3 Measurements

#### 3.3.1 Tolerance of uncertainty

The Stress from Uncertainty (SUS) measures the tolerance of uncertainty on issues pertaining solely to clinical uncertainty (Gerrity et al., 1990). It represents the stress resulting from uncertainty, such as anxiety, uneasiness, discomfort, and emotional turmoil. The SUS is derived from responses to a continuous 0–100 Likert scale questionnaire consisting of 12 questions with answers ranging from “Extremely Unlikely” to “Extremely Likely.” The higher the score, the higher the stress. The Cronbach's alpha of 0.9002 suggests a high degree of internal consistency. The mean SUS score for our sample is 50.08 (SD = 18.2), and the summary statistics of the SUS are shown in Table 3.

**TABLE 3:** Summary statistics for the measurements.

		frequency	%	cum.%
Nightingale	Risk averse	44	46.32	46.32
	Prospect theory discordant	10	10.53	56.84
	Prospect theory concordant	27	28.42	85.26
	Risk seeking	14	14.74	100.00
SUS (mean)	0–25	8	8.33	8.33
	26–50	36	37.50	45.83
	51–75	42	43.75	89.58
	76–100	10	10.42	100.00
Regret (mean)	0–25	5	5.21	5.21
	26–50	23	23.96	29.17
	51–75	61	63.54	92.71
	76–100	7	7.29	100.00

#### 3.3.2 Regret

The scale developed by Schwartz et al. (2002) was selected to measure the individual's tendency to experience regret in our study. The scale was designed to identify individuals who chronically exhibit abnormally higher (and lower) levels of tendency to experience regret. The regret scale was constructed from the answers to a 100‐point Likert scale questionnaire consisting of five questions about regret in real‐life decisions. The higher the measure, the higher the tendency to feel regret when making decisions (Table 3). The estimated Cronbach's alpha is 0.5572 for the regret scale, which is suggestive of marginal reliability. The mean regret score is 55.76 (SD = 14.91).

#### 3.3.3 Risk preference

Nightingale risk preference instrument (1987) was selected for our measure of revealed risk preference. It was applied in the analysis of physicians' decisions, including the decisions to hospitalize patients (Nightingale, 1988), making recommendation for medical procedures (Nightingale & Grant, 1988), and medical testing (Zaat & van Eijk, 1992).

The Nightingale risk preference instrument was designed to elicit choices between risky and certain options for patient outcomes, both for the gain and the loss domains. Based on their responses to these two questions, the respondents were categorized into four groups: “risk‐averse” if they chose the certain option in both questions (choice of options A&A), “risk‐seeking” if they chose the risky option in both questions (B&B), “prospect theory concordant” if they chose the risky option for the loss scenario and certain option in gain scenario (A&B), and “prospect theory discordant” if they chose the risky option for the gain scenario and certain option in loss scenario (B&A). “Prospect theory concordant” indicates that individuals see losses as more significant than similar gains, leading them to prefer risky options in loss situations, whereas “prospect theory discordant” posits the contrary.

The first group (A&A) is the largest of these four categories (46.32%), followed by the prospect theory concordant category (28.42%), then by the risk‐seeking category (14.74%), and finally the prospect theory discordant category (10.53%) shown in Table 3.

## 4 RESULT

### 4.1 Measurements

#### 4.1.1 Tolerance of uncertainty

To explore the effects of individual characteristics on the SUS, we estimated a linear regression with the SUS score as the dependent variable and respondent characteristics including gender, years of experience (or age), ethnicity, position, number of operations per year, health status (Table 4). The experience, number of operations per year, age, gender, ethnicity, and health status variables are not significant in explaining the regret score. However, trainees were more likely to experience higher scores on stress under uncertainty compared to the consultant (Table 4, columns 10–14).

**TABLE 4:** OLS regression of SUS mean on respondent characteristics.

Dependent variables	SUS_mean													
	(1)	(2)	(3)	(4)	(5)	(6)	(7)	(8)	(9)	(10)	(11)	(12)	(13)	(14)
Years of experience	−0.173	−1.249^*^				−0.140	−1.310^*^				0.233	0.645		
	(0.195)	(0.681)				(0.192)	(0.746)				(0.250)	(0.962)		
Experience squared		0.029					0.031^*^					−0.009		
		(0.017)					(0.019)					(0.023)		
Age			−0.106	−2.363				−0.042	−2.700				0.158	1.565
			(0.192)	(1.756)				(0.192)	(1.921)				(0.242)	(2.184)
Age squared				0.024					0.028					−0.014
				(0.018)					(0.020)					(0.022)
Female	1.704	2.052	1.821	2.256	3.223	2.868	3.566	3.476	4.317	0.713	1.011	0.757	1.012	0.498
	(4.221)	(4.267)	(4.240)	(4.235)	(3.950)	(4.240)	(4.394)	(4.299)	(4.414)	(3.818)	(4.026)	(4.059)	(4.004)	(3.954)
Asian/Asian British	0.299	−1.201	0.670	−0.580	3.017	2.022	0.491	2.541	1.284	−1.691	−2.000	−1.759	−2.046	−1.706
	(5.543)	(5.874)	(5.645)	(5.836)	(5.300)	(5.605)	(5.983)	(5.717)	(5.969)	(4.758)	(5.046)	(5.009)	(5.067)	(5.053)
Mixed/Other ethnic group	0.960	2.101	0.591	1.690	3.077	3.093	4.071	3.006	4.097	2.101	2.642	2.538	1.150	0.713
	(5.861)	(5.814)	(6.500)	(6.426)	(6.549)	(6.580)	(6.326)	(7.133)	(6.848)	(4.568)	(4.557)	(4.579)	(4.822)	(4.829)
So‐so	5.748	5.637	5.396	4.867	5.775	5.954	5.551	5.762	4.808	5.428	3.832	4.004	4.580	5.114
	(8.041)	(7.825)	(8.036)	(7.777)	(6.953)	(7.182)	(7.078)	(7.269)	(6.937)	(7.336)	(7.618)	(7.606)	(7.462)	(7.457)
Healthy	−6.699	−6.636	−7.417	−7.345	−5.706	−5.117	−5.340	−5.356	−5.582	−6.487	−6.998	−6.938	−6.882	−6.720
	(6.933)	(6.551)	(7.056)	(6.688)	(6.433)	(6.293)	(6.093)	(6.515)	(6.046)	(6.396)	(6.457)	(6.481)	(6.504)	(6.526)
Very healthy	−11.028	−10.941	−11.067	−10.900	−11.475	−11.769	−12.021	−11.545	−11.836	−8.822	−8.124	−7.781	−7.947	−7.405
	(8.281)	(7.782)	(8.391)	(7.937)	(7.980)	(7.782)	(7.311)	(7.976)	(7.370)	(7.382)	(7.354)	(7.353)	(7.490)	(7.470)
Operations: 101–250					3.773	4.757	5.945	3.848	6.094					
					(7.048)	(7.654)	(8.160)	(7.667)	(8.663)					
Operations: 251–500					9.307	10.498	12.245	11.488	13.836					
					(7.090)	(7.589)	(8.106)	(7.546)	(8.361)					
Operations: Over 500					0.803	2.371	4.674	2.065	4.599					
					(8.124)	(8.818)	(9.706)	(8.984)	(10.015)					
Trainees										13.726^***^	17.814^***^	20.091^***^	15.457^***^	18.515^**^
										(4.326)	(5.646)	(6.990)	(5.780)	(7.138)
Other										17.627^***^	17.988^***^	18.074^***^	17.344^***^	18.034^***^
										(6.072)	(6.489)	(6.425)	(6.285)	(6.156)
Constant	55.750^***^	62.583^***^	57.997^***^	108.799^***^	46.589^***^	47.610^***^	53.646^***^	46.930^***^	104.776^**^	48.441^***^	43.708^***^	39.830^***^	40.944^***^	6.700
	(6.550)	(7.031)	(10.210)	(40.095)	(8.874)	(8.923)	(7.865)	(11.439)	(40.883)	(6.067)	(7.623)	(10.518)	(13.118)	(53.710)
*N*	91	91	88	88	93	91	91	88	88	92	90	90	87	87

*Note*: Standard errors in parentheses.

Operations: numbers of operations in a year.

Other: participants are neither consultants nor trainees.

Coefficient reported shows the average predicted probability.

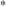
 p<0.1; 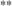 p<0.05; 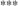 p<0.01.

#### 4.1.2 Regret

To explore the effects of individual characteristics on regret, we estimated a linear regression with the regret score as the dependent variable and respondent characteristics (Table 5). The experience, gender, ethnicity, and health status variables are not significant in explaining the regret score. In contrast, years of experience and age are significantly negatively associated with regret. The number of operations per year is a significant predictor of the regret score, which reveals a reverse U‐shape relationship shown in the estimation, and the trainees and others are more likely to experience regret than the consultant.

**TABLE 5:** OLS regression of Regret mean on respondent characteristics.

Dependent variables	Regret_mean													
	(1)	(2)	(3)	(4)	(5)	(6)	(7)	(8)	(9)	(10)	(11)	(12)	(13)	(14)
Years of experience	−0.376^**^	−0.841^*^				−0.344^**^	−0.853^*^				−0.220	−0.163		
	(0.148)	(0.491)				(0.147)	(0.481)				(0.215)	(0.813)		
Experience squared		0.013					0.014					−0.001		
		(0.013)					(0.013)					(0.018)		
Age			−0.330^**^	−1.063				−0.304^**^	−1.489				−0.229	0.863
			(0.143)	(1.235)				(0.144)	(1.186)				(0.211)	(1.877)
Age squared				0.008					0.013					−0.011
				(0.013)					(0.013)					(0.018)
Female	−2.644	−2.494	−2.975	−2.834	−1.231	−2.090	−1.786	−2.372	−1.997	−2.958	−2.985	−3.020	−3.397	−3.796
	(3.164)	(3.244)	(3.221)	(3.263)	(3.163)	(3.222)	(3.365)	(3.416)	(3.508)	(3.003)	(3.109)	(3.189)	(3.144)	(3.154)
Asian/Asian British	3.351	2.703	4.179	3.774	7.546^*^	4.997	4.330	5.663	5.103	3.429	2.480	2.513	3.053	3.316
	(3.863)	(3.807)	(3.914)	(3.984)	(4.059)	(4.114)	(4.063)	(4.180)	(4.193)	(3.580)	(3.790)	(3.786)	(3.879)	(3.891)
Mixed/Other ethnic group	8.228^**^	8.720^**^	9.396^**^	9.753^**^	9.035^**^	8.628^*^	9.054^*^	9.485^*^	9.972^**^	9.342^**^	8.939^**^	8.924^**^	9.652^**^	9.313^**^
	(3.850)	(4.055)	(4.264)	(4.406)	(4.317)	(4.516)	(4.693)	(4.804)	(4.925)	(3.723)	(3.767)	(3.793)	(4.008)	(4.084)
So‐so	2.748	2.700	1.606	1.435	−0.238	2.131	1.956	0.954	0.529	1.138	1.686	1.710	0.896	1.311
	(7.767)	(8.043)	(7.856)	(8.015)	(6.332)	(7.031)	(7.383)	(7.121)	(7.441)	(8.476)	(8.518)	(8.599)	(8.621)	(8.672)
Healthy	−2.811	−2.784	−3.927	−3.903	−4.684	−3.173	−3.270	−4.049	−4.150	−3.767	−3.102	−3.093	−3.930	−3.804
	(7.353)	(7.647)	(7.475)	(7.667)	(6.009)	(6.411)	(6.796)	(6.566)	(6.870)	(8.114)	(8.066)	(8.119)	(8.203)	(8.202)
Very healthy	4.058	4.096	3.301	3.355	2.646	2.247	2.138	1.749	1.619	5.718	5.204	5.251	4.602	5.023
	(8.225)	(8.470)	(8.473)	(8.633)	(7.617)	(7.720)	(8.041)	(7.990)	(8.226)	(8.695)	(8.775)	(8.929)	(9.042)	(9.061)
Operations: 101–250					10.551^**^	12.084^**^	12.602^**^	10.993^**^	11.994^**^					
					(5.113)	(4.918)	(4.991)	(5.127)	(5.477)					
Operations: 251–500					9.049^*^	10.129^**^	10.890^**^	9.465^*^	10.512^*^					
					(5.180)	(5.085)	(5.219)	(5.363)	(5.662)					
Operations: Over 500					3.483	5.705	6.708	5.339	6.468					
					(5.822)	(5.591)	(5.792)	(5.802)	(6.132)					
Trainees										10.334^***^	7.104	7.417	6.569	8.942
										(3.694)	(5.169)	(7.051)	(5.354)	(7.524)
Other										8.105^**^	8.621^***^	8.632^***^	9.394^***^	9.929^***^
										(3.328)	(3.065)	(3.077)	(3.233)	(3.305)
Constant	61.906^***^	64.857^***^	71.239^***^	87.722^***^	48.993^***^	52.781^***^	55.410^***^	62.002^***^	87.783^***^	53.090^***^	57.203^***^	56.670^***^	64.468^***^	37.902
	(6.845)	(7.313)	(8.728)	(27.571)	(6.858)	(7.093)	(7.410)	(9.604)	(26.115)	(7.830)	(9.170)	(11.935)	(13.416)	(48.247)
*N*	91	91	88	88	93	91	91	88	88	92	90	90	87	87

*Note*: Standard errors in parentheses.

Operations: numbers of operations in a year.

Other: participants are neither consultants nor trainees.

Coefficient reported shows the average predicted probability.

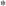
 p<0.1; 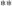 p<0.05; 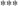 p<0.01.

#### 4.1.3 Risk preference

To explore the effects of individual characteristics on risk preference, we ran a multivariate logit regression of the Nightingale instrument on the demographic characteristics including gender, years of experience (or age), ethnicity, position, number of operations per year, health status, and found that none of these characteristics is a significant collective predictor of risk preferences, except healthy individuals are more likely to be predicted as risk‐seeking than participants with poor health (Table 6). 

**TABLE 6:** Nightingale mlogit regression marginal effect.

Dependent variables	Averse	PTD	PTC	Seeking	Averse	PTD	PTC	Seeking	Averse	PTD	PTC	Seeking	Averse	PTD	PTC	Seeking
	(1)	(2)	(3)	(4)	(5)	(6)	(7)	(8)	(9)	(10)	(11)	(12)	(13)	(14)	(15)	(16)
Age	0.010	0.001	−0.009	−0.003					−0.123^*^	0.100	0.046	−0.022				
	(0.007)	(0.005)	(0.006)	(0.006)					(0.065)	(0.063)	(0.056)	(0.047)				
Female	0.005	0.070	−0.025	−0.050	0.028	0.055	−0.032	−0.051	0.045	0.045	−0.044	−0.047	0.057	0.027	−0.044	−0.040
	(0.114)	(0.073)	(0.099)	(0.097)	(0.115)	(0.070)	(0.099)	(0.099)	(0.108)	(0.059)	(0.093)	(0.097)	(0.113)	(0.060)	(0.095)	(0.100)
Trainees	0.050	−0.102	−0.135	0.187	0.087	−0.085	−0.131	0.129	−0.188	0.076	−0.030	0.142	−0.114	0.156	−0.061	0.018
	(0.180)	(0.097)	(0.113)	(0.162)	(0.180)	(0.099)	(0.117)	(0.170)	(0.198)	(0.210)	(0.177)	(0.197)	(0.206)	(0.204)	(0.156)	(0.163)
Other	−0.176	−0.158^***^	0.018	0.316^*^	−0.148	−0.146^***^	−0.024	0.318^*^	−0.235	−0.126^***^	0.035	0.327^*^	−0.188	−0.122^***^	−0.033	0.343^**^
	(0.152)	(0.050)	(0.152)	(0.175)	(0.155)	(0.048)	(0.144)	(0.176)	(0.159)	(0.037)	(0.140)	(0.177)	(0.155)	(0.035)	(0.124)	(0.164)
Asian/Asian British	−0.090	0.085	−0.028	0.032	−0.093	0.090	−0.036	0.040	−0.088	0.076	−0.025	0.037	−0.110	0.096	−0.034	0.048
	(0.146)	(0.140)	(0.133)	(0.092)	(0.144)	(0.138)	(0.136)	(0.091)	(0.150)	(0.119)	(0.130)	(0.092)	(0.147)	(0.115)	(0.126)	(0.095)
Mixed/Other ethnic group	−0.019	0.091	−0.027	−0.044	0.037	0.064	−0.046	−0.054	0.029	0.058	−0.045	−0.041	0.054	0.047	−0.045	−0.056
	(0.178)	(0.111)	(0.156)	(0.115)	(0.175)	(0.105)	(0.149)	(0.108)	(0.171)	(0.090)	(0.148)	(0.117)	(0.164)	(0.089)	(0.144)	(0.096)
So‐so	0.278	−0.162	−0.198	0.082^*^	0.251	−0.177	−0.159	0.085^*^	0.219	−0.127	−0.171	0.079^*^	0.243	−0.153	−0.165	0.074^**^
	(0.207)	(0.147)	(0.236)	(0.046)	(0.211)	(0.144)	(0.237)	(0.048)	(0.214)	(0.133)	(0.243)	(0.044)	(0.211)	(0.126)	(0.237)	(0.038)
Healthy	0.232	−0.103	−0.306	0.177^***^	0.260	−0.131	−0.310	0.182^***^	0.194	−0.068	−0.305	0.179^***^	0.246	−0.105	−0.331^*^	0.190^***^
	(0.190)	(0.146)	(0.208)	(0.051)	(0.187)	(0.140)	(0.200)	(0.047)	(0.190)	(0.134)	(0.211)	(0.028)	(0.187)	(0.124)	(0.201)	(0.047)
Very healthy	0.220	0.004	−0.428^*^	0.204	0.199	0.013	−0.408^*^	0.196	0.150	0.059	−0.413^*^	0.205	0.174	0.051	−0.407^*^	0.182^*^
	(0.226)	(0.171)	(0.219)	(0.131)	(0.222)	(0.166)	(0.209)	(0.120)	(0.217)	(0.148)	(0.225)	(0.126)	(0.206)	(0.137)	(0.213)	(0.096)
Operations: 101−250	0.032	−0.192	−0.006	0.166^*^	0.032	−0.198	−0.005	0.170^*^	0.082	−0.173	−0.083	0.173^**^	0.045	−0.185	−0.055	0.195^**^
	(0.222)	(0.150)	(0.191)	(0.094)	(0.214)	(0.148)	(0.190)	(0.090)	(0.241)	(0.159)	(0.195)	(0.080)	(0.228)	(0.169)	(0.185)	(0.076)
Operations: 251−500	0.222	−0.066	−0.135	−0.021	0.244	−0.092	−0.133	−0.019	0.259	−0.040	−0.200	−0.019	0.263	−0.079	−0.181	−0.002
	(0.229)	(0.175)	(0.196)	(0.092)	(0.221)	(0.164)	(0.191)	(0.103)	(0.238)	(0.179)	(0.194)	(0.081)	(0.233)	(0.180)	(0.184)	(0.076)
Operations: Over 500	0.198	−0.056	−0.254	0.111	0.222	−0.074	−0.260	0.111	0.248	−0.031	−0.328^*^	0.111	0.249	−0.073	−0.307	0.131
	(0.241)	(0.178)	(0.189)	(0.114)	(0.234)	(0.176)	(0.190)	(0.118)	(0.255)	(0.183)	(0.193)	(0.103)	(0.248)	(0.188)	(0.188)	(0.099)
Years of experience					0.010	0.002	−0.006	−0.006					−0.028	0.044^*^	0.011	−0.027
					(0.007)	(0.004)	(0.007)	(0.008)					(0.030)	(0.024)	(0.023)	(0.025)
Age squared									0.001^**^	−0.001	−0.001	0.000				
									(0.001)	(0.001)	(0.001)	(0.000)				
Experience squared													0.001	−0.001	−0.000	0.001
													(0.001)	(0.001)	(0.001)	(0.001)
*N*	87	87	87	87	90	90	90	90	87	87	87	87	90	90	90	90

*Note*: Standard errors in parentheses.

Averse: risk averse; PTD: prospect theory discordant; PTC: prospect theory concordant; Seeking: risk seeking.

Operations: numbers of operations in a year.

Other: participants are neither consultants nor trainees.

Coefficient reported shows the average predicted probability.

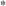
 p<0.1; 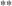 p<0.05; 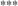 p<0.01.

### 4.2 Willingness to proceed

The average willingness to proceed for low‐, medium‐, and high‐risk scenarios is 88 (SD = 18.61), 76 (SD = 21.44), and 79 (SD = 31.52), respectively (see Table 7).

**TABLE 7:** Summary statistics of willing to serve in three scenarios.

	mean	min	max	sd	count
WTP_Low_risk	88	15	100	18.61	96
WTP_Medium_risk	76	10	100	21.44	97
WTP_High_risk	79	0	100	31.52	97

We examine the effect of risk preference, tolerance of uncertainty, and regret attitudes on anesthetists' WTP in each risky scenario using a nonparametric kernel model and the estimation results are reported in Table 8. Our choice of the model is based on its suitability to the data structure and the fact that it imposes very few assumptions on the relationship between the dependent variable and the covariates. We do not have a priori information to assume a specific functional form, for instance, we do not have sufficient information to assume a linear functional form. One of our objectives is to discover the relationship between the dependent and independent variables. In addition, there is little support for making a specific distributional assumption about the noise term. The following equation is applied in the regression,

(1) $${\text{WTP}}_{i,s}=m\left(X_{i,s}\right)+\varepsilon_{i,s},\;i=1,\text{…},96\;s=1,2,3,$$

where ${\text{WTP}}_{i,s}$ captures the operational response in each scenario s from individual i, and $X_{i,s}$ is a vector of several variables including SUS score, regret score, risk preference, scenario risk, years of experience, gender, position, ethnicity, health status, correctly identified scenario risk, and number of operations in a year.

**TABLE 8:** Non‐parametric kernel regression.

Dependent variables	WTS								
	(1)	(2)	(3)	(4)	(5)	(6)	(7)	(8)	(9)
Mean									
WTS	80.919^***^	81.785^***^	80.777^***^	81.082^***^	80.205^***^	80.662^***^	80.825^***^	80.924^***^	81.705^***^
	(1.527)	(1.482)	(1.620)	(1.493)	(1.597)	(1.659)	(1.648)	(1.537)	(1.634)
Effect									
SUS mean score		−0.220^***^				−0.190^**^			−0.191^*^
		(0.071)				(0.089)			(0.112)
Regret mean score			−0.320^**^				−0.309^**^		−0.188
			(0.136)				(0.145)		(0.162)
Prospect theory discordant				0.139				0.386	2.667
				(0.178)				(1.259)	(1.816)
Prospect theory concordant				0.147				0.061	2.845
				(0.269)				(2.521)	(3.545)
Risk seeking				−0.110				0.110	5.104
				(0.210)				(3.501)	(5.189)
Medium risk	−10.396^***^	−9.169^***^	−7.280^***^	−10.940^***^	−9.469^***^	−6.846^***^	−6.082^***^	−6.271^***^	−5.709^***^
	(2.352)	(1.982)	(1.789)	(2.435)	(1.832)	(1.706)	(1.795)	(1.639)	(1.896)
High risk	−8.207^***^	−9.784^***^	−10.089^***^	−8.270^**^	−9.691^***^	−9.027^***^	−7.596^**^	−8.032^**^	−7.062^*^
	(2.925)	(3.573)	(3.751)	(3.225)	(3.349)	(3.502)	(3.593)	(3.343)	(3.791)
Years of experience					0.309	0.333^*^	0.166	0.230	0.276
					(0.195)	(0.199)	(0.199)	(0.201)	(0.221)
Female					−11.333^***^	−12.894^***^	−12.239^***^	−10.296^***^	−14.380^***^
					(2.751)	(3.453)	(3.163)	(2.638)	(4.473)
*N*	290	287	281	284	272	266	257	269	248
Control	No	No	No	No	Yes	Yes	Yes	Yes	Yes

*Note*: Control variables: Position, Ethnicity, Lifestyle, Number of operations.

Bootstrap standard errors with 500 replications.

Standard errors in parentheses.

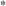
 p<0.1; 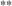 p<0.05; 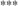 p<0.01.

The predicted average of WTP is about 80 across the scenarios and individuals. The participants are predicted 6% less likely to WTP in the medium‐risk scenario compared to the low‐risk scenario and 9% less likely in the high‐risk scenario compared to the low‐risk scenario after controlling for SUS mean score, regret mean score, and risk preference, respectively (Table 8, columns 6 and 7). This indicates that participants, on average, have a good understanding of the scenario risk.

**FIGURE 1:**
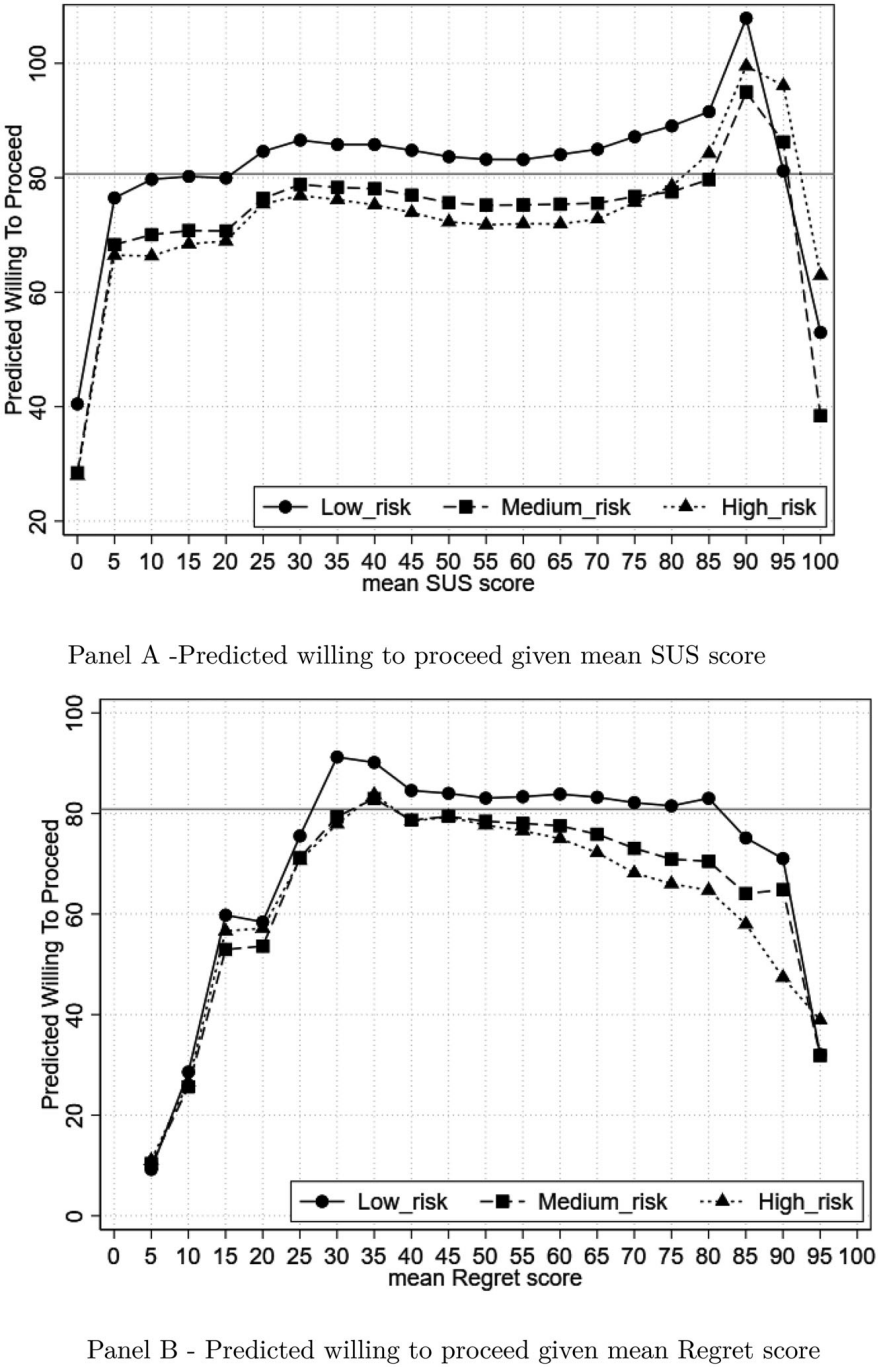
Predicted willing to proceed.

The SUS mean score is a significant predictor of WTP, and the estimation is robust against the inclusion of various combinations of right‐hand‐side variables (Table 8, columns 2, 6, and 9) which supports hypothesis 1. Thus, the more stress the clinical uncertainty causes to the anesthetist, the less likely they are to agree to take part in the procedure. It is predicted that a 10‐point increase in the SUS score leads to a 2.2 percentage point decrease in the WTP. Figure 1, Panel A, shows the predicted WTP across the SUS score for each risky scenario where the red line indicates the predicted mean of WTP using the estimation result in Table 8, column 6. The effect of SUS score is relatively consistent in the three scenarios.

Our data indicate that regret has a significant adverse effect on the WTP (Table 8, columns 3 and 7) which supports hypothesis 2. The negative marginal effect suggests that as a participant's tendency to experience regret increases, they are less likely to agree to proceed in the procedure. It is predicted that a 10‐point increase in the regret score leads to a 3.1 percentage point decrease in the WTP, on average. Figure 1, Panel B, shows the predicted across the regret score for each risky scenario where the horizontal line indicates the predicted mean of WTP using the estimation result in Table 8, column 7. The relationship between the regret scores and the predicted WTP resembles an inverted U‐shape. For individuals scoring between 5 and 30, an increase in regret score leads to an increase in WTP. A further increase in regret scores leads to a decline in WTP. In addition, the effect of regret on WTP is not uniform across the three scenarios. There is a stronger reduction in WTP in the medium‐ and high‐risk scenarios compared to the low‐risk scenario given that participants score greater than 50 on the regret measure.

The individual Nightingale measures of risk preferences are not significant regardless of whether demographic controls are included. Thus, there are no statistically significant differences between risk‐averse participants (baseline) and participants from each of the other three risk‐attitude categories, as a result, we did not find enough evidence to support hypothesis 3.

Finally, when all three individual trait measures are included in the regression (see Table 8, column 9), the SUS coefficient is still significant, and the direction of the regret coefficient is negative, which is consistent with the previous estimates, though the significance of coefficients is weakened which is likely due to the correlation between measurements (see Appendix A). Overall, the estimation results yield strong evidence that individual‐level psychological traits play a key role in expert clinical decisions.

**FIGURE 2:**
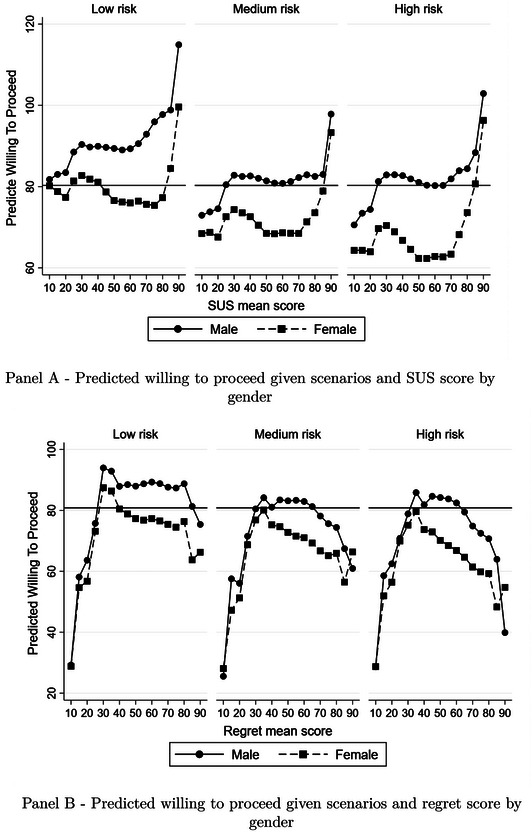
Predicted willing to proceed by gender.

Next, we examined the heterogeneity in the prediction of WTP by gender. The earlier research has demonstrated significant gender differences in anesthetist training (Pearce et al., 2020), but no studies have been conducted to determine whether gender differences exist in the profession in general. There are two main observations. First, the trend of SUS and regret impact on WTP is similar between males and females, and the differences (i.e., the gap at each level of SUS and regret, respectively) remain relatively constant across scenarios at all levels of SUS and regret score (see Figure 2 and Panel A and Panel B, respectively). This suggests the gender effect does not vary by levels of SUS or regret. Second, the overall effect of gender is highly significant in that men are on average 10 points more likely to indicate WTP than women (see Table 8, columns 5–9), thus supports hypothesis 4. This finding is consistent with earlier gender research on decision‐making showing that men are more likely to engage in risky activities than women (Filippin & Crosetto, 2016). Both observations conclude that the gender differences are not filtered through by SUS and regret, but directly at the decision‐making stage.

Finally, we included a control dummy variable “Correctly identify” in our regression to capture participants' understanding of the scenario risk level. The variable is constructed by comparing participant's responses to the risk level they perceived in a question with the actual risk ranking. There are no significant differences in the WTP between the participants who correctly identified the scenario risks and those who did it incorrectly. None of the remaining controls, including years of experience (no evidence is found to support hypothesis 5), position, ethnicity, health status, and number of operations per year, is significant.

## 5 DISCUSSION

Understanding healthcare professionals' clinical decision‐making is paramount to improving clinical and training practice. To date, despite serving a key role within the operating theater and elsewhere in hospitals, there has been little data on what factors impact anesthetists' willingness to proceed as attending anesthetists. Previous work has shown variations in WTP but has largely failed to examine what factors could help explain these variations. Our study was designed to augment and expand these earlier investigations. Our results show, first, a similar variation in WTP in scenarios characterized by varying levels of surgical risks. Moreover, we can provide insight into the mechanisms driving these variations, by elucidating the effect of risk preference, tolerance of uncertainty, and sensitivity of regret on the variation in the anesthetists' likelihood to attend operations.

A growing body of research has highlighted the importance of tolerance of uncertainty, risk tolerance, and the tendency to experience regret in clinical decision‐making and education. Drawing on this line of work, our results indicate that individual differences in tolerance of uncertainty, tendency to experience regret, and gender contribute to the explanation of individual differences in WTP. First, we found that a higher tolerance of uncertainty predicts a higher WTP in all scenarios, regardless of the level of risk (low, medium, or high). These results mirror and support earlier work showing that stress due to uncertainty negatively impacts a wide range of therapeutic judgments and practices (Bursztajn, 1981; Fox, 1980; Schneider et al., 2010). Our data is also aligned with previous studies that illustrate that high tolerance of uncertainty is linked to selecting a better course of action (Fox, 1980), and, more importantly, with a previous systematic review (Strout et al., 2018), showing that low tolerance of uncertainty is also linked to low tolerance of risk.

Second, an inverse U‐shape was found between the tendency to experience regret and WTP. A positive correlation has been observed between the tendency to experience regret and the willingness to engage in decision‐making, with the anticipated effect exhibiting a reversal at a score of 30 (out of 100). Hence, the average effect of regret is negative in predicting the likelihood of WTP. The significance of regret is consistent with prior studies that have demonstrated its influence on medical decision‐making (Becerra Pérez et al., 2016; Richner et al., 2017; Wilson et al., 2017). In addition, our findings indicate that the impact of regret on operational decisions is not universally consistent across varying levels of tendency to experience regret. The negative effect of regret is increasingly pronounced as the scenarios' risk level increases. For individuals who scored high levels of regret scores (score>50), a higher regret score was associated with a significant decrease in WTP in a high‐risk scenario compared to the lower‐risk scenarios. This is likely because higher‐risk scenarios tend to have a greater probability of leading to adverse outcomes.

Third, our findings demonstrate that the negative relationship between being female, and some operational decisions is both robust and statistically significant. Female anesthetists were less likely to proceed regardless of the level of risk presented in the three scenarios, regret, and attitudes toward uncertainty. In addition, while the gender effect is not significant in predicting risk preferences, regret, and tolerance of uncertainty, it is significant in the decision‐making stage—the decision to act as the attending anesthetist. Our findings, however, should not be interpreted as implying that women's clinical judgments are less desirable or effective than those of men. To clarify, we do not make any judgment about which gender's clinical profile leads to better decisions. Instead, our results align with previous research highlighting gender‐based differences in areas such as pain management and decisions regarding cesarean delivery. Our work suggests that females may exhibit tendencies toward underconfidence, while males may lean toward overconfidence. Importantly, there are clinical situations where a more conservative approach might be advantageous and others where a riskier approach could be preferable. We believe that clinical training should address and balance these tendencies, as has been recommended in contexts like pain management and cesarean delivery decision‐making. Although there may not be a significant difference in anesthesiology educational attainment based on gender, there is evidence to suggest that decision‐making processes vary between males and females, implying that education in the form of more practical training alone cannot address gender differences. A possible explanation would be the differences in confidence level. Previous studies (Pearce et al., 2020; Sidhu & Civil, 2019) have found gender effects in anesthesia training resulting from the confidence gap between males and females. Despite the accumulation of years of experience, our findings indicate that a gender disparity persists among consultants (more senior members of anesthetists). Thus, simply increasing the number of practices in the training is unlikely to be enough to reduce the gender variation. In the early stages of anesthetist clinical education, it is important to integrate a greater understanding of why such disparities exist, as well as continuous support programs to assist female anesthetists in overcoming the confidence gap.

Last but not least, our result shows an interesting not significant effect of risk preference and experience on the WTP. First, the insignificance of risk preference may be due to the fact that medical professionals (GPs) tend to minimize the risks they are willing to take when treating patients (Grol et al., 1990), thereby minimizing the distinctions between risk‐preference groups. Also, Harrison et al. (2005) have argued that the instrument used to measure risk could impact the results. Thus, our measure of risk preference might not be sensitive enough to predict clinical decisions. Future studies, thus, might need to include other measures of risk‐taking propensities.

In addition, we found that years of experience were not a significant predictor of WTP. We measure experience along three dimensions: years of experience, number of operations in a year, and position of being a consultant (senior anesthetist) or a trainee. Our data revealed no strong correlations between the three variables, and none of the measures were significant in predicting clinical decisions. Previous work, likewise, has reported mixed results. A study with British anesthetists (Greig et al., 2017), for example, found that experience was possibly linked to variation in response, while a similar study among Saudi anesthetists (Alkassimi et al., 2020) failed to find a correlation. Our results further indicate that experience does not necessarily help explain disparities in clinical decisions.

Several limitations should be acknowledged. First, like previous studies in the field, our data collection method was based on what respondents might do and not necessarily what they will do. While survey‐based studies are useful and valid, their abilities to predict real‐life decisions and behavior are always questionable. Future studies could examine these questions in the clinical setting, but the challenges and practicality of collecting data in vivo render this methodology extremely hard. The wide spectrum of responses, however, provides us with confidence that participants provided honest and meaningful answers. Moreover, our data might not be representative as participants in our study were recruited from only two hospitals. This issue is endemic to many studies investigating healthcare professionals' decisions. Our sample, moreover, was drawn from UK participants only, and as such, we are unable to judge its generalizability to other healthcare systems. Thus, replications of our results in different healthcare systems are needed. It should be noted, moreover, that 16% of those who started the study did not complete the survey. As the survey was mostly completed during working hours, it is possible that work‐related demands could help explain the rate of attrition. As our final sample closely represents the population of anesthetists in the UK, we do not believe this represents a major concern. Finally, while the scenarios used in this study were developed and validated by highly experienced anesthetists, they only represent a limited spectrum of clinical scenarios anesthetists face in clinical practice.

Individual variation in clinical practice represents one of the key challenges faced by modern medicine and is driven to a large extent by physicians' decisions. Anesthetists are not immune to this phenomenon, so gaining insight into the factors underlying variations is key. Developing educational and training programs can help minimize inappropriate decision variation, including improving self‐awareness of personal traits and making appropriate adjustments. To our knowledge, at present, there are no bespoke programs designed to educate and train anesthetics trainees to better deal with uncertainty and regret. Researchers, however, have explored several educational paths (Moulder et al., 2022; Papanagnou et al., 2021)—such as team debrief, role modeling, simulations, and storytelling—that could support and improve medical trainees' capacity to deal and manage uncertainty. Others (Moffett et al., 2023) have explored the use of a digital educational escape room to help medical trainees learn how to better deal with uncertainty. These represent some promising educational interventions to help physicians better cope with uncertainty, which can be incorporated in the anesthetics teaching curriculum. Our findings shed light on the importance of tolerance of uncertainty, regret, and gender in affecting operational decision‐making, which we believe would be useful in improving both clinical practice and education for future generations of anesthetists.

## Figures and Tables

**FIGURE A1:**
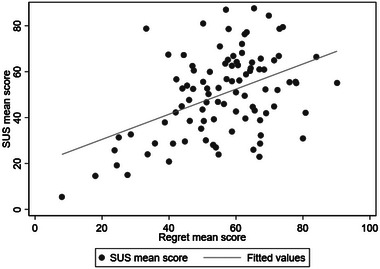
Scatter‐plot of mean SUS score and mean Regret score.

**FIGURE A2:**
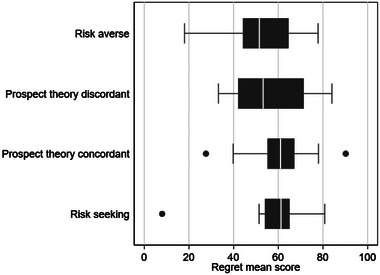
Box‐plot of mean regret score condition on Nightingale instrument.

**FIGURE A3:**
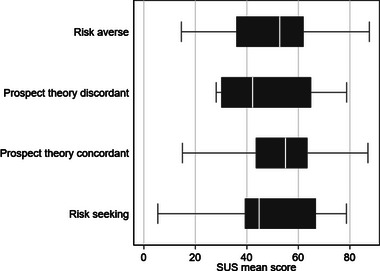
Box‐plot of mean SUS score condition on Nightingale instrument.

## APPENDIX A MEASUREMENT SCALES

We compared the three measurement scales in this session.

A positive correlation was observed between intolerance of uncertainty and regret in the past. Djulbegovic et al. (2015) find a strong positive correlation between intolerance of uncertainty and regret attitude among physicians. A significant correlation was also observed in our study. The correlation coefficient between the SUS score and the regret score is 0.448 (Figure A1).

Previous literature has shown a positive correlation between risk aversion and regret, while the relationship between risk aversion and intolerance has produced inconsistent results. The experiment conducted by Lauriola et al. (2019) demonstrates that decision‐makers who are more likely to take regret feelings into account were more risk‐averse than those who did not consider regret as much. Carleton et al. (2016) suggested that increasing uncertainty intolerance is associated with increased risk‐averse behavior, on the other hand, Allen et al. (2011) show no association between risk tolerance and uncertainty tolerance when evaluating medical decision conditions on clinical type, including physician, nurse, practitioner, and physician assistant, and Andruchow et al. (2012) show no association between risk tolerance and uncertainty tolerance in resource utilization for emergency department trauma patients.

Our analysis revealed statistically significant variations in the median regret score for participants with different risk preferences. Individuals who are risk‐averse obtained regret scores that were lower compared to individuals who are risk‐seeking (Figure A2). This result is consistent with previous findings (Lauriola et al., 2019) that risk‐averse individuals take regret feelings into account so that they have a lower tendency to feel regret. However, no statistically significant differences were observed in the SUS score, as determined through the nonparametric Kruskal–Wallis rank test (Figure A3).
